# Performance of FACSPresto Point-of-Care Instrument for CD4-T Cell Enumeration in Human Immunodeficiency Virus (HIV)-Infected Patients Attending Care and Treatment Clinics in Belgium and Tanzania

**DOI:** 10.1371/journal.pone.0170248

**Published:** 2017-01-27

**Authors:** Géraldine Daneau, Said Aboud, Irena Prat, Willy Urassa, Luc Kestens

**Affiliations:** 1 Laboratory of Immunology, Department of Biomedical Sciences, Institute of Tropical Medicine, Antwerp, Belgium; 2 Department of Microbiology and Immunology, Muhimbili University of Health and Allied Sciences (MUHAS) Dar es Salaam, Tanzania; 3 World Health Organisation (WHO), Geneva, Switzerland; 4 Department of Biomedical Sciences, University of Antwerp, Antwerp, Belgium; Universita degli Studi di Roma Tor Vergata, ITALY

## Abstract

**Background:**

CD4 T-cell counts are widely used to assess treatment eligibility and to follow-up HIV-infected patients. The World Health Organization prequalification of *in vitro* diagnostics program conducted a performance evaluation of the FACSPresto (BD Biosciences), a new point-of-care instrument to measure absolute CD4-T cell (CD4) counts and percentages in venous and capillary blood samples from HIV-infected patients.

**Methods:**

Patients were recruited in Belgium (200 patients) and in Tanzania (247 patients). Venous blood samples were analyzed in two nearby reference laboratories. In addition, nurses/technicians collected a capillary blood sample by finger prick directly into a FACSPresto CD4 cartridge. Assay precision was assessed on fresh blood and on external quality control samples. Trueness (bias) was assessed by comparing results from FACSPresto with the reference (single-platform FACSCalibur). Clinical misclassification was measured at 200, 350 and 500 cells/μL thresholds.

**Results:**

Intra-assay precision was < 6%, and inter-assay < 8%. CD4 results from FACSPresto and reference method resulted in regression slopes of 0.99–1.11 using either venous or capillary blood. Correlation was better for venous than for capillary blood (minimum 0.97 vs 0.93 respectively). Capillary blood resulted in a larger bias than venous blood, with 24 and 83 cells/μL for absolute CD4 counts on capillary blood in Antwerp and Dar es Salaam respectively, vs 12 and 41 cells/μL on venous blood. Bias on CD4% was < 1% on both venous and capillary blood, and was proportionally better than for absolute CD4 counts. Clinical misclassification was in line with the average overestimation, showing a very good specificity, but sensitivity around 70–90%. The rejection rate was 11% on first reading, leading to 6% of all samples without final result after a second reading.

**Conclusions:**

The FACSPresto performed very well on venous blood samples, and well on capillary blood samples.

## Introduction

In 2014, there were about 36.9 million people living with HIV [1]. Of these, 32.5 million were eligible for antiretroviral treatment (ART) but, in low- and middle-income countries, only 36% received treatment [2]. ART initiation was for long based on absolute CD4 T-cell counts (CD4 count) apart from clinical examination. Currently, the World Health Organization (WHO) recommends starting treatment for all HIV-infected patients irrespective of CD4 count levels, with priority for patients with severe or advanced HIV clinical disease (WHO clinical stage 3 or 4) and adults with less than 350 cells/μL [3]. The previous guidelines mentioned a threshold of 500 cells/μL, which could be seen as an intermediary step towards treatment for all in settings where the threshold of 350 cells/μL to initiate ART is still applied [4]. Even when the importance of CD4 counts to monitor ART is decreasing, it is still recommended to monitor CD4 counts at regular time intervals until stable viral load suppression is achieved [5;6]. In addition, as long as viral load testing is difficult due to technical and financial constraints, it is expected that low-income regions will continue using CD4 counts as an alternative [7]. CD4 counts are also used to decide on prophylaxis against opportunistic infections, for instance to screen for cryptococcal infection in patients with ≤ 100 CD4 cells/μL and stop prophylaxis when patient presents with > 200 cells/μL, or start co-trimoxazole in case CD4 counts are ≤ 350 cells/μL [3]. The threshold of 200 cells/μL is also important to decide on vaccination benefits and risks [3].

Clinical management of HIV-infected patients is therefore still dependant on CD4 counting capacity. The CD4 reference method, developed on large flow cytometers, is usually implemented by skilled staff in well-equipped laboratories [8;9]. In the last decade, several instruments dedicated to CD4 counting were developed. They are easier to operate and cheaper than the traditional flow cytometers used in clinical laboratories [10]. However, this new generation of instruments still require a laboratory infrastructure and trained staff. Simple CD4 technologies at the point of care (POC) could fill a last gap by bringing CD4 counting to small health centres or as instruments operated in mobile teams [11]. Having a CD4 technology nearby the patient with a short turn-around time for results would help to reduce the number of patients lost to follow up [12]. Currently, there is no instrument-free rapid test for CD4 enumeration. All POC CD4 technologies still require an instrument; in this case, a reader.

Before being widely distributed, manufacturer-independent evaluations of new technologies are valuable to confirm the appropriate use of such technologies in different settings. In this perspective, the WHO prequalification of *in vitro* diagnostics program includes the evaluation of CD4 counting instruments, in particular those meant for use in resource-limited settings. The FACSCount CD4 and the Pima CD4 were successfully evaluated by WHO in 2012 [13]. In 2014, WHO evaluated a new CD4 POC instrument, the FACSPresto. This instrument presents similar features to the Pima CD4 but, in addition to CD4 counts, also measures CD4 percentages (CD4%) and haemoglobin concentration. The 25μl-blood drop from the patient is inserted in a ready-to-use disposable cartridge, and incubated on the bench during 18 minutes prior to analysis. The CD4 counting process itself, in the FACSPresto instrument, is fully automated with multiple internal control checks, and results are available within 3–4 minutes.

This study analyses the analytical performance of this recent POC instrument in two different settings, comparing CD4 counting results in Europe and in Africa. In accordance with the WHO protocol, analytical precision and trueness of FACSPresto were measured in two independent reference labs, in Belgium and in Tanzania. This is one of the first publications on the performance of FACSPresto, and the first manufacturer-independent reporting of paired comparison of venous and capillary blood samples in a reference setting [14–17].

## Materials and Methods

### Study populations

Adult patients presenting for routine CD4 enumeration at the HIV outpatient clinic at the Institute of Tropical Medicine (ITM) Antwerp, Belgium (in April-June 2014), and at the Infectious Diseases Clinic (IDC) in Dar es Salaam, Tanzania (in June 2014), were invited to donate a capillary blood sample (by a single finger prick) in addition to routine venipuncture in K_3_-EDTA vacutainer tubes. After a first phase of recruiting all consecutive patients, a second phase focused on patients with low CD4 counts, based on measurement at their previous visit, in order to reach target numbers per CD4 category.

The study was approved by the Institutional Review Board from ITM and the Ethical committee at University Hospital Antwerp, Belgium, and by the Senate Research and Publications Committee of Muhimbili University of Health and Allied Sciences in Dar es Salaam, Tanzania. All patients signed an informed consent form prior to the inclusion in the study.

#### Analytical precision

Intra-laboratory variation testing included instrument precision, intra-assay variation, inter-instrument variation, and inter-assay variation, using venous blood specimens. Capillary blood specimens were only used to assess instrument precision. Both sites had multiple FACSPresto instruments to facilitate work management (four FACSPresto instruments in Antwerp and three in Dar es Salaam); the variation between those instruments was measured (inter-instrument precision).

The mean, standard deviation (SD) and coefficient of variation (% CV) of the obtained results were calculated and compared between FACSPresto instruments (Chi-square).

In Antwerp, blood samples were stratified in categories around clinically relevant CD4 thresholds: 200 (150–250), 350 (300–400) and 500 (450–550) CD4 cells/μL. The CD4 category was determined by the first CD4 result of venous blood sample on the FACSPresto, except for instrument precision studies which was based on capillary blood results. Precision analysis in Dar es Salaam mainly focused on low CD4 samples, i.e. values between 100 and 300 CD4 cells/μL.

Instrument precision (run-to-run) consisted of re-reading a single CD4 cartridge ten times on the same FACSPresto instrument. The corresponding venous blood was processed in the same way. Only when the venous blood sample was included in the same CD4 category as capillary blood, precision results were included in the study.

For intra-assay precision (tube-to-tube variability), each blood sample was prepared to fill ten cartridges, and each cartridge was read once, with all ten cartridges on the same FACSPresto instrument.

For inter-instrument precision (instrument-to-instrument variability), each sample was prepared to fill ten cartridges, and each cartridge was read four times, once on each of the four FACSPresto instruments in Antwerp, by the same operator. In Dar es Salaam, each sample was prepared to fill ten cartridges, by each of the three operators, and each cartridge was read on each of the three FACSPresto instruments. The average of the obtained results was calculated for each instrument, and for each operator. The inter-instrument variability (%CV inter-instruments) was calculated using the average results of the different instruments for each patient sample. The reproducibility (in Tanzania) was calculated between the averages of the different operators and instruments for each sample, and compared with a one-way ANOVA.

For inter-assay precision (day-to-day variability), a venous blood sample was analysed within 2h after blood collection. An aliquot of the venous blood sample was kept at room temperature and prepared for FACSPresto at two additional time points to include 6 and 24 hours after blood collection.

Global mean %CV for each category was calculated as a weighted average of the individual sample %CV’s.

#### Quality control

The CD4 reference method implemented in Antwerp is ISO15189 certified, and in Dar es Salaam by the National AIDS Control Program in the Ministry of Health, Community Development, Gender, Elderly and Children in Tanzania. Both laboratories participate in external quality assessment programs (QASI and UK-NEQAS). Prior to the start of the study, the CD4 reference instruments (FACSCalibur) used in both settings were technically inspected by a service engineer from BD Biosciences.

During the study, the CD4 reference instruments were calibrated daily, and checked by running Multicheck Normal and Low controls (BD Biosciences). Three different lots of Normal controls and two lots Low controls were used during the three-month duration of the study in Antwerp. In Dar es Salaam, only one lot of Normal controls was used for the one-month study.

The FACSPresto contains internal control checks which are run each time the instrument is powered up. In addition, CD-Chex Plus BC Normal and Low (Streck, Omaha, NE) were run daily for two months on each FACSPresto instrument in Antwerp, and for the whole one-month study in Dar es Salaam (the same lot was used thorough the study).

For each blood control sample, the reported results were compared to the sample validation range provided by the manufacturer. The mean, SD and %CV of the obtained results were calculated and compared between FACSPresto instruments and/or operator.

#### Trueness

Venous and capillary blood samples were collected by three nurses in Antwerp, and prepared (for venous blood samples) then read on FACSPresto by one technician on one of the four FACSPresto instruments. Blood samples in Dar es Salaam were collected and prepared by three technicians; each technician used a single FACSPresto instrument for reading.

CD4 counts and CD4% measured on FACSPresto using venous and capillary blood were compared to the corresponding CD4 venous blood results on FACSCalibur (BD Biosciences, San Jose, CA). CD4 counts were measured according to manufacturer's recommendations using Multiset reagents (CD3/CD4/CD8/CD45 monoclonal antibodies) and BD Trucount tubes (both from BD Biosciences, San Jose, CA). Analysis on FACSCalibur was done manually using CellQuest software using the accredited procedure used for routine CD4 counting in the laboratories.

Agreement between FACSPresto results and those obtained by the reference method, and between venous and capillary blood results obtained on FACSPresto, was assessed by the Passing and Bablok regression analysis [18], correlation with Spearman’s ρ coefficient, Bland-Altman [19] and Pollock analysis [20] using MedCalc version 10.0.2.0 (MedCalc Software, Mariakerke, Belgium). Outliers were identified and removed from the statistical analysis when the values for absolute and relative bias were both larger than four times the respective mean bias [21]. Correlation coefficient and bias were compared between sites and between parameters (blood types, CD4 vs CD4%, and influencing factors), applying paired or independent t-test, or one-way ANOVA as required.

Clinical misclassification was calculated for CD4 counts at clinically relevant CD4 thresholds of 200, 350, 500 cells/μL, using the reference method results as the “true” value for sensitivity and specificity. Inter-rater agreement was calculated with the Kappa coefficient [22].

### Invalid results (Rejection rate)

Invalid results or sample rejection was defined as the inability of FACSPresto instrument to provide CD4 results from a patient blood sample. In case of an invalid test result on a capillary blood sample, it was not possible to redraw a second capillary blood specimen. As the study was based on four (Antwerp) or three (Dar es Salaam) different FACSPresto instruments in the participating labs, the cartridge was read on another FACSPresto instrument when rejected once or twice (repeated rejection). In case of a rejected first reading on venous blood, a second reading was performed on the same cartridge. If that the sample was rejected again, a new cartridge was prepared and read multiple times when required (after rejection). Venous and capillary blood samples from the same patient were always read on the same FACSPresto instrument.

## Results

### Study population

In Antwerp, 200 patients were recruited. Clinical data was available for 198 patients, of whom 197 were HIV infected. The median age (minimum—maximum) of the study participants was 46 years (19–83), and 71% were male. Most patients (96%) were on anti-retroviral therapy (ART). The median CD4 count was 420 cells/μL; 18 had CD4 < 200 cells/μL (median 158 cells/μL), 108 had CD4 counts between 200–500 cells/μL (351 cells/μL), and 73 had CD4 > 500 cells/μL (658 cells/μL). None of the patients presented malaria; three patients had suspected tuberculosis, but were finally sputum and/or culture negative.

In Dar es Salaam, 247 patients were recruited. The median age (minimum—maximum) was 41 years (15–75) and 32% were male. Most patients (93%) received ART. The median CD4 cell count was 266 cells/μL (n = 245); 82 had CD4 < 200 cells/μL (median 123 cells/μL), 114 had a CD4 count between 200–500 cells/μL (301 cells/μL), and 49 had CD4 > 500 cells/μL (636 cells/μL). Two patients had suspected malaria, one of these had suspected tuberculosis too.

### Analytical precision

The mean instrument precision (%CV) for absolute CD4 counts and percentages in capillary and venous blood was <3%. The intra-assay variability showed an overall %CV of 5.9% for both absolute CD4 counts and CD4%. The %CV (intra-assay) for the paired samples was similar between the four FACSPresto instruments tested in Antwerp, and between the three instruments tested in Dar es Salaam (p > 0.05 for each site), and the inter-instrument variability was < 2%. The operator had no impact on the %CV (p > 0.05) (S1 Table). Finally, the inter-assay variability presented larger %CV, but still < 10% on average. Values for the different CD4 categories for each precision assay are presented in Table 1.

**Table 1:** Coefficient of variation (%CV) for the different precisions assays.

Site and category (CD4/μL)	n	Instrument (capillary)		Instrument (venous)		Intra-assay (venous)		Inter-assay (venous)		Inter-instrument (venous)		Reproducibility (venous)	
		CD4	CD4%	CD4	CD4%	CD4	CD4%	CD4	CD4%	CD4	CD4%	CD4	CD4%
**Antwerp**	**12**^**$**^	1.5	1.4	2.3	1.9	4.9	4.3	7.3	5.7	1.3	1.1	-	-
**150–250**	4*	1.7	1.6	3.0	2.7	6.1	5.0	11.7	7.7	2.5	2.1	-	-
**300–400**	4*	1.2	1.1	1.5	1.2	5.2	4.3	6.6	7.7	0.5	0.6	-	-
**450–550**	4*	1.5	1.4	2.4	1.7	3.5	3.6	3.6	1.8	0.7	0.4	-	-
**Dar es Salaam**	**10**							6.8	7.5				
**100–300**	**10**^**$#**^	3.4	2.9	3.5	4.0	7.0	7.9	7.5	8.5	-	-	-	-
**301–500**	**2–3**^**#**^	-	-	-	-	-	-	5,0	5.3	3.3	2.7	4.3	3.7
**600–800**	**2**	-	-	-	-	-	-	-	-	0.7	0.8	0.8	1.6
**Mean**		2.5	2.1	2.9	3.0	5.9	5.9	7.0	6.7	1.5	1.3	2.6	2.7

Mean precision (%CV, coefficient of variation) in Antwerp and in Dar es Salaam, for different CD4 categories. Number of samples (n), except for instrument precision with n = 15 (^$^), with 5 in each category (*), and for inter-assay in Antwerp with n = 3 per category (*), and in Dar es Salaam (^#^) with n = 7 for 100–300 CD4/μL, and 3 for 301–500 CD4/μL. For inter-instrument, and for reproducibility in Dar es Salaam, n = 2 in each category (none for 100–300 in Dar es Salaam).

### Quality control

All internal controls passed on the FACSPresto. Mean %CV in Antwerp was 0.14% for Normal and 0.16% for Low control. Mean %CV in Dar es Salaam was 0.07% for Normal and 0.11% for Low control. Values for the different FACSPresto instruments are presented in S2 Table.

Inter-assay variability based on commercially available stabilized blood samples CD-Chex Plus BC external controls in Antwerp showed globally %CVs < 10%; for CD4%: 2.1% for Normal and 6.2% for Low values, and for absolute CD4: 3.1% for Normal and 7.8% for Low values.

In Dar es Salaam, the global %CV was also <10%; for CD4%: 2.9% for Normal and 4.7% for Low values, and for absolute CD4 counts: 3.0% for Normal and 8.9% for Low.

All Multicheck controls analysed on FACSCalibur with Trucount tubes passed. In Antwerp, inter-assay variabilities (%CV) were 1.8% for Normal and 5.0% for Low for CD4 percentages. For absolute CD4 counts, the mean %CV was 6.7% for Normal and 4.9% for Low. Detailed results on different batches are presented in S2 Table. Values in Dar es Salaam for Normal were 16% for absolute CD4 counts and 4.0% for CD4%.

### Trueness

Agreement between FACSPresto and the reference methods are shown in Table 2 (Venous blood on FACSPresto versus reference), Table 3 (Capillary blood on FACSPresto versus venous blood on reference), and Table 4 (Capillary blood versus venous blood on FACSPresto). Passing Bablok regression analyses showed slopes ranging from 0.99–1.11 for all comparisons, in both Antwerp and Dar es Salaam. The correlation coefficient was significantly higher when using venous blood compared to capillary blood (p < 0.01). The correlation coefficient was also different between sites, and was better (higher) on CD4% when comparing FACSPresto to FACSCalibur in Dar es Salaam (p < 0.05).

**Table 2:** FACSPresto performance with venous blood on FACSPresto compared to FACSCalibur (reference).

	Antwerp			Dar es Salaam		
	n	CD4 cells/μL	CD4%	n	CD4 cells/μL	CD4%
**Regression**	196^$^	y = 0.23+1.03x	y = 0.37+0.99x	230^$^	y = 8,72+1,11x	y = 0,02+1,03x
**(95% CI slope)**		(1.00;1.05)	(0.97;1.01)		(1.08;1.14)	(1.01;1.04)
**Correlation R**	196^$^	0.983	0.985	230^$^	0.974 *	0.993 ***
**Absolute bias**		cells/μL	%		cells/μL	%
**mean bias on all (LOA)**	196^$^	12 (-63;+87)	0.1 (-2.7;+3.0)	230^$^	41 (-64;+146) ***	0.4 (-2.0;+2.8) *
**on low CD4**	16	5 (-28;+38)	-0.2 (-1.8;+1.4)	75	27 (-30;+83)	0.0 (-2.2;+2.3)
**on middle CD4**	108	13 (-54;+81)	0.2 (-3.1;+3.4)	110	50 (-58;+158)	0.5 (-2.0;+3.1)
**on high CD4**	72^$^	12 (-79;+103)	0.2 (-2.1;+2.5)	45^$^	45 (-100;+189)	0.8 (-1.2;+2.8)
**Relative bias**		%	%		%	%
**mean bias on all (LOA)**	196^$^	2.5 (-15;+20)	0.6 (-11;+13)	230^$^	15 (-17;+46) ***	2.6 (-16;+21) *
**on low CD4**	16	0.4 (-24;+25)	-2.5 (-16;+11)	75	20 (-17;+57)	2.5 (-26;+31)
**on middle CD4**	108	3.3 (-16;+22)	1.0 (-13;+15)	110	14 (-15;+43)	2.7 (-11;+16)
**on high CD4**	72^$^	1.9 (-10;+14)	0.7 (-6.4;+7.9)	45^$^	6.3 (-15;+28)	2.8 (-4.0;+9.6)

Regression with Passing-Bablok analysis, Correlation with Spearman’s ρ coefficient, absolute bias with Bland-Altman analysis, relative bias with Pollock analysis. Due to outlier exclusion,

^$^ n+1 for CD4%.

LOA: limits of agreement. Low CD4 ≤ 200 cells/μL, middle 201–500 cells/μL, high CD4 > 500 cells/μL.

* for p-value < 0.05,

** p < 0.01,

*** p < 0.001 to compare mentioned parameter between sites (Dar es Salaam vs Antwerp).

**Table 3:** FACSPresto performance with capillary blood on FACSPresto compared to FACSCalibur (reference).

	Antwerp			Dar es Salaam		
	n	CD4 cells/μL	CD4%	n	CD4 cells/μL	CD4%
**Regression**	193^$^	y = 0.55+1.04x	y = 0.07+1.03x	224^&^	y = 9.88+1,25x	y = -0,29+1,01x
**(95% CI slope)**		(1.00;1.08)	(1.00;1.06)		(1.20;1.29)	(0.99;1.03)
**Correlation R**	193^$^	0.966^###^	0.973^##^	224^&^	0.939 ** ^##^	0.988 *** ^###^
**Absolute bias**		cells/μL	%		cells/μL	%
**mean bias on all (LOA)**	193^$^	24 (-122;+169) ^#^	0.9 (-3.1;+4.8) ^###^	224^&^	83 (-97;+262) *** ^###^	-0.3 (-3.2;+2.7) *** ^###^
**on low CD4**	16	15 (-34;+63)	-0.1 (-2.3;+2.1)	76^$^	43 (-25;111)	-0.5 (-3.5;+2.5)
**on middle CD4**	105^$^	21 (-79;+121)	0.8 (-3.3;+4.8)	104^$^	100 (-49;+249)	-0.3 (-3.4;+2.8)
**on high CD4**	72	29 (-176;+235)	1.2 (-2.7;+5.1)	44	109 (-188;+407)	0.1 (-2.4;+2.6)
**Relative bias**		%	%		%	%
**mean bias on all (LOA)**	193^$^	4.7 (-26;+35) ^##^	3.2 (-13;+20) ^###^	224^&^	26 (-12;+63) *** ^###^	-2.7 (-29;+23) *** ^###^
**on low CD4**	16	6.3 (-35;+48)	-2.0 (-27;+23)	76^$^	31 (-14;+75)	-5.6 (-45;+33)
**on middle CD4**	105^$^	4.9 (-26;+36)	3.4 (-13;+20)	104^$^	26 (-5.1;+57)	-1.9 (-19;+15)
**on high CD4**	72	4.1 (-22;+30)	4.0 (-8.9;+17)	44	16 (-18;+49)	0.4 (-8.5;+9.4)

Regression with Passing-Bablok analysis, Correlation with Spearman’s ρ coefficient, absolute bias with Bland-Altman analysis, relative bias with Pollock analysis. Due to outlier exclusion,

^$^ n-2 for CD4%;

^&^ n-4 for CD4%.

LOA: limits of agreement. Low CD4 ≤ 200 cells/μL, middle 201–500 cells/μL, high CD4 > 500 cells/μL.

* for p-value < 0.05,

** p < 0.01,

*** p < 0.001 to compare mentioned parameter between sites (Dar es Salaam vs Antwerp).

^#^ for p-value < 0.05,

^##^ p < 0.01,

^###^ p < 0.001 to compare mentioned parameter with Venous blood performance (Table 3 versus Table 2).

**Table 4:** FACSPresto performance with venous and capillary blood on FACSPresto, compared to each other’s.

	Antwerp			Dar es Salaam		
	n	CD4 cells/μL	CD4%	n	CD4 cells/μL	CD4%
**Regression**	193	y = 4.00+1.00x	y = -0.50+1.04x	217^$^	y = -1,19+1,12x	y = -0,32+0,98x
**(95% CI slope)**		(0.97;1.04)	(1.01;1.07)		(1.09;1.15)	(0.96;1.00)
**Correlation R**	193	0.967^###^	0.974^##^	217^$^	0.980 *	0.986 ** ^###^
**Absolute bias**		cells/μL	%		cells/μL	%
**mean bias on all (LOA)**	193	11 (-121;+143)	0.7 (-3.2;+4.6) ^##^	217^$^	44 (-66;+154) ***	-0.7 (-4.0;+2.5) *** ^###^
**on low CD4**	16	10 (-50;+69)	0.1 (-2.8;+3.0)	71^$^	16 (-41;+73)	-0.4 (-3.4;+2.6)
**on middle CD4**	105	10 (-88;+107)	0.5 (-3.4;+4.4)	103	49 (-64;+161)	-1.0 (-4.6;+2.6)
**on high CD4**	72	14 (-166;+193)	1.1 (-2.9;+5.0)	43	79 (-49;+206)	-0.7 (-3.6;2.2)
**Relative bias**		%	%		%	%
**mean bias on all (LOA)**	192	3.1 (-30;+36)	3.0 (-21;+27) ^#^	217^$^	11 (-21;+43) *** ^#^	-5.4 (-31;+20) *** ^###^
**on low CD4**	16	5.7 (-40;+51)	0.5 (-25;+26)	71^$^	9.7 (-36;+55)	-7.5 (-46;+31)
**on middle CD4**	105	3.7 (-33;+41)	3.2 (-25;+32)	103	12 (-13;+36)	-5.2 (-22;+11)
**on high CD4**	72	1.7 (-22;+25)	3.3 (-9.4;+16)	42	11 (-6.6;+28)	-2.3 (-12;+7.4)

Regression with Passing-Bablok analysis, Correlation with Spearman’s ρ coefficient, absolute bias with Bland-Altman analysis, relative bias with Pollock analysis. Due to outlier exclusion,

^$^ n-1 for CD4%.

LOA: limits of agreement. Low CD4 ≤ 200 cells/μL, middle 201–500 cells/μL, high CD4 > 500 cells/μL.

* for p-value < 0.05,

** p < 0.01,

*** p < 0.001 to compare mentioned parameter between sites (Dar es Salaam vs Antwerp).

^#^ for p-value < 0.05,

^##^ p < 0.01,

^###^ p < 0.001 to compare mentioned parameter with venous blood versus reference (Table 4 versus Table 2).

**Fig 1:**
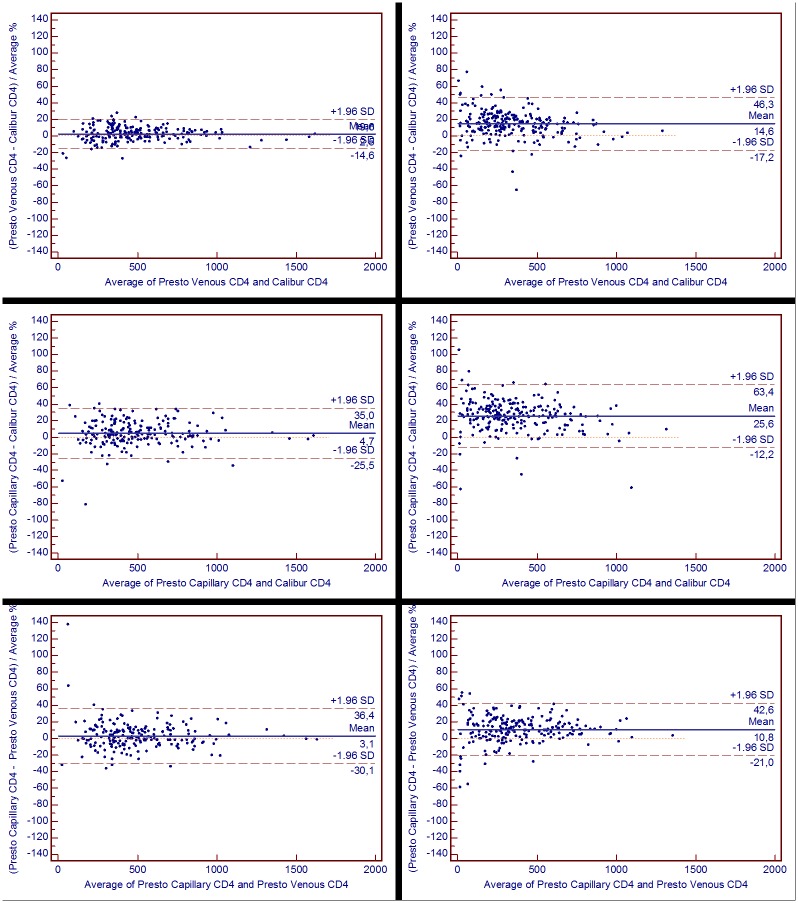
Pollock analysis for absolute CD4 counts. Relative bias data (%) from Antwerp (left) and Dar es Salaam (right), with CD4 results (cells/μL) obtained by FACSPresto (Presto) using venous (upper) and capillary blood (midden), each compared to reference (Calibur), and to one another’ (lower). Mean bias is represented by the horizontal solid blue line, with the long-dashed brown line for limits of agreement (= 1.96SD), each mentioned with respective value.

**Fig 2:**
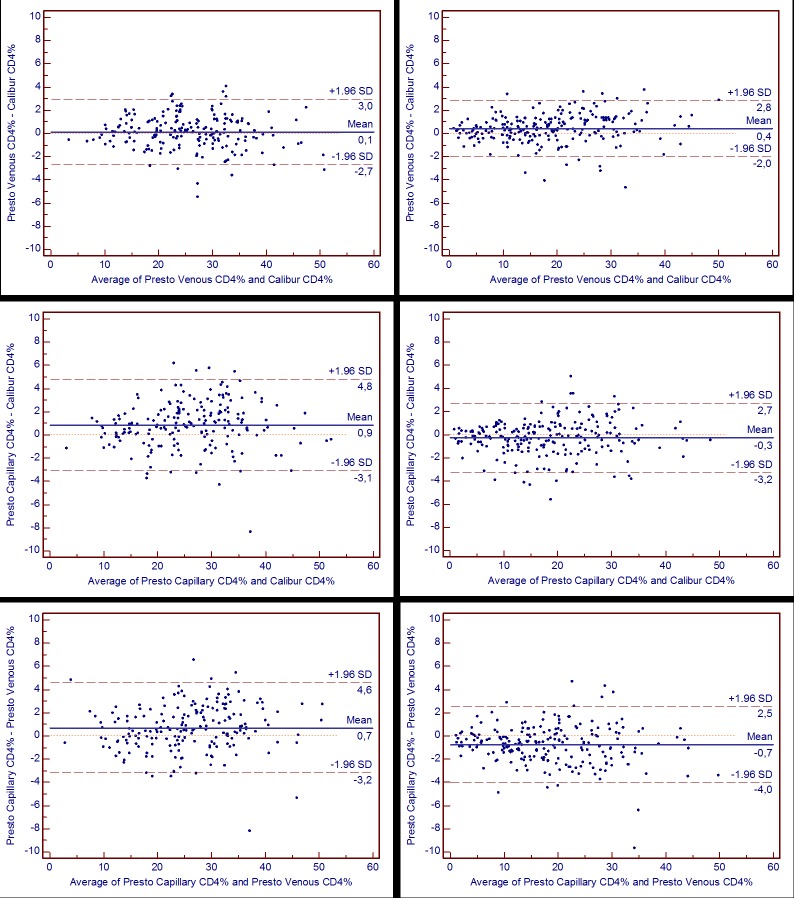
Bland-Altman analysis for CD4%. Absolute bias data (%) from Antwerp (left) and Dar es Salaam (right), with value on FACSPresto (Presto) using venous (upper) and capillary blood (midden), each compared to reference (Calibur), and compared to each others’ (lower). Mean bias is represented by the horizontal solid blue line, with the long-dashed brown for limits of agreement (= 1.96SD), each mentioned with respective value.

In Antwerp, the comparison of absolute CD4 counts in venous blood resulted in a mean absolute bias of +12 cells/μL between FACSpresto and FACSCalibur with limits of agreement up to +/- 87 cells/μL (1.96 x SD) around the mean bias. This comes down to a relative bias of 2.5%, with 1.96 x SD of 20% (Fig 1, upper left). Using capillary blood, the bias almost doubled (Fig 1, middle left). In Dar es Salaam the observed bias also doubled using capillary instead of venous blood (Fig 1, right). The absolute bias on CD4 percentages was also different between venous and capillary blood (each compared to the reference) (Table 3 and Fig 2). On the other hand, the absolute bias between venous blood and capillary blood (both on FACSPresto) was similar to the bias observed between capillary blood (on FACSPresto) and reference (venous blood on FACSCalibur) for absolute CD4 counts (Table 4 and Fig 1 lower). Similarly, performance on CD4 percentages on both venous and capillary blood comparison was different between sites (Tables 2, 3 and 4). In addition, the relative bias on CD4 counts in Dar es Salaam was significantly worse than on CD4% (p < 0.001; Tables 2, 3 and 4). In Antwerp, this difference was only observed for relative bias on FACSPresto (venous or capillary blood) vs reference (p < 0.001 and < 0.05 respectively; Tables 2 and 3).

We analyzed external factors that may influence the bias. Nurses collecting capillary blood in Antwerp had a significant impact on the observed bias (p = 0.028). On the contrary, operator in Dar es Salaam (technician collecting blood and measuring on FACSPresto device) had no influence (p > 0.05). Similarly, the different FACSPresto instruments produced a similar bias (p > 0.05).

### Clinical misclassification

Misclassification analysis is shown in Table 5. Kappa coefficient was 0.642 to 0.893 for the different thresholds (200, 350 and 500 cells/μL). Specificity was in all cases higher than 90%. Sensitivity, however, was lower, in particular at the 200 and 350 thresholds. A misclassification means that the clinician will not start ART at the expected time. To have a broader idea of the time delay, bias between FACSPresto and FACSCalibur for misclassified samples is shown in Table 6. The larger the bias, the longer the expected time delay; samples with < 50 cells/μl may be expected to have no large impact on treatment timing despite the misclassification.

**Table 5:** Clinical misclassification of FACSPresto for venous and capillary blood versus FACSCalibur, at strict thresholds ≤ 200, 350 and 500 cells/μL.

Decision	*At 200 cells/μL*		*At 350 cells/μL*				*At 500 cells/μL*			
	Venous blood	Capillary blood	Venous blood		Capillary blood		Venous blood		Capillary blood	
	Dar es Salaam	Dar es Salaam	Antwerp	Dar es Salaam	Antwerp	Dar es Salaam	Antwerp	Dar es Salaam	Antwerp	Dar es Salaam
**n**	230	224	196	230	193	224	196	230	193	224
**% disease**	33	33	36	63	36	63	67	80	63	79
**kappa**	0.746	0.642	0.761	0.767	0.770	0.643	0.893	0.643	0.819	0.568
**Sensitivity**	71	57	77	84	79	72	93	85	89	79
**Specificity**	99	100	96	97	96	98	99	92	96	98

Clinical misclassification was calculated for CD4 counts at clinically relevant CD4 thresholds of 200, 350, 500 cells/μL, using the reference method results as the “true” value for disease diagnostics. Inter-rater agreement was calculated with the Kappa coefficient.

**Table 6:** Absolute bias (cells/μL) between FACSPresto and FACSCalibur in misclassified samples at 350 cells/μL.

	*ART started earlier*				*ART started later*			
	Venous blood		Capillary blood		Venous blood		Capillary blood	
	Antwerp	Dar es Salaam	Antwerp	Dar es Salaam	Antwerp	Dar es Salaam	Antwerp	Dar es Salaam
**n**	5	3	5	2	15	16	13	31
**Mean bias**	-35	-152	-49	-139	46	89	65	121
**SD**	6	90	37	61	26	40	36	41
**Min**	-26	-64	-13	-96	3	24	9	37
**Max**	-42	-243	-102	-182	104	200	128	232
**Bias 50–100 (n)**	0	1	1	1	5	11	7	9
**Bias > 100 (n)**	0	2	1	1	1	3	2	21

### Invalid results (Rejection rate)

Invalid results on FACSPresto tend to occur more frequently with venous blood than with capillary blood specimens (11.0% versus 7.4% of agreement samples at the first reading; p = 0.08) (S3 Table). Re-reading a rejected capillary blood cartridge was not always sufficient to give a valid result, leading to 6% of patients not receiving a CD4 value. Using venous blood where new cartridges could be prepared, 5.8% of the blood samples did not give valid CD4 results. Most rejection codes were linked to an instrument failure (82% and 53% on capillary and venous blood respectively) (S4 Table). The frequency of invalid results was similar for all seven FACSPresto instruments (p > 0.05). In addition, three blood specimens failed to produce results due to very low values (< 20 cells/μl on FACSCalibur); they were considered as a valid reading on the FACSPresto.

### Operators’ feed-back

Technicians found FACSPresto instrument easy to use after a 4-hour training.

Nurses in Antwerp found taking finger prick blood impractical and preferred taking blood by venipuncture. They found the success of taking capillary blood variable and too dependent on hand temperature. When hands are cold, despite manual warming, it was really hard to get a good blood drop, especially without squeezing the punctured finger. In contrast, warm hands tended to bleed too hard, requiring protective clothes, and spilling blood on the floor or on the cartridge itself, despite the frame around collector. Moreover, contact with HIV-infected blood was closer than with venous blood, requiring use of gloves for nurse safety.

Similarly, technicians in Dar es Salaam found the FASPresto easy to use. However, venous blood is routinely practiced and technicians preferred venous blood compared to capillary blood.

## Discussion

The performance evaluation of the FACSPresto was conducted simultaneously in two complementary sites selected by WHO: one in Antwerp, Belgium, and the other in Dar es Salaam, Tanzania. Both study sites collected venous and capillary blood samples from each patient, which were analysed in the respective reference labs, with high quality assurance to ensure good working conditions.

The FACSPresto was found to perform well for the different parameters. Intra-assay precision was on average < 6%, which was as good as our reference instruments (data not shown). Inter-assay analysis, with repeat measurements at different time points, is used to assess repeatability of the test results. The instructions for use of FACSPresto recommend to use fresh blood samples or aged blood of 24h maximum, which does not allow day-to-day variability assays on stabilized blood covering more than 24h. We performed three repeated measurements on fresh blood samples, with two time points on the first day and an additional time point at 24h. In addition, we used stabilized blood samples (CD-Chex Plus BC quality controls) to measure day-to-day variability over a period of 1–3 months. The results confirm our observations on fresh blood samples. Furthermore, the precision on fresh blood and controls was found to be similar to that observed in the previous evaluations of the FACSPresto [14–17].

The trueness (bias) of venous blood results on FACSPresto was assessed by comparison with the FACSCalibur (reference). We found a slight overestimation of the absolute counts, similar to the one observed for other CD4-dedicated bench top instruments like the FACSCount [13]. Results from Dar es Salaam were also perfectly in line with FACSPresto results obtained in two other studies recently conducted in Africa [15;16]. Correlation and clinical misclassification of patients based on capillary blood results were also in agreement with the previous studies [14–16]. An additional element in our study was the analysis of factors influencing final result, with identification of capillary blood collection, and the nurse performing the finger prick. Lower performance on capillary blood samples as compared to venous blood illustrates that the type of blood specimen can have a substantial impact on results. In Dar es Salaam, CD4 percentages were quite similar between FACSPresto and FACSCalibur. However, there was less agreement between absolute CD4 counts, in capillary blood in particular. This raises the concern of the contribution of the observed bias as a consequence of possible lack of accuracy of the reference results. Absolute CD4 counting is based on a single platform measurement using Trucount tubes and precision of pipetting is very critical. The high CV on Multicheck Normal controls in Dar es Salaam is consistent with such variability from the reference; some outlier values within the batch could either be due to pipetting or heterogeneous sample. Unfortunately, no third instrument was included to confirm the patient results of the reference instrument. In addition, up to four outliers were excluded from the statistical analysis. As this approach is justified from a statistical point of view, exclusion of the FACSCalibur or FACSPresto results would probably not happen in the clinical practice. The patient would therefore be monitored according to the “outlier” result.

Concerning the statistical analyses included in our article, we applied a relative bias on both absolute CD4 counts and on CD4 percentages. Relative bias is usually not used on CD4%. However, this approach allowed us to compare bias estimates between different groups and between absolute CD4 counts and CD4 percentages, which showed a significant difference in our evaluation. Additionally, we applied the Pollock analysis after exclusion of samples with < 100 CD4 cells/μL, to avoid impact of a high relative bias at low CD4 counts (data not shown) on the overall average bias on all CD4 counts. This exclusion method did not reduce the LOA on relative bias by more than 1%.

The misclassification of patients for treatment initiation (prophylaxis or ART) was higher when capillary blood results were used compared to venous blood. The sensitivity was also affected by the threshold level, with better performance at higher thresholds. This may be partially explained by the sample distribution, as less samples had low CD4 counts, so the 24 missclassified samples represented a high proportion of the 82 samples with ≤ 200 cells/μL. We did not apply the threshold of 200 cells/μL on the CD4 results from Antwerp, as only 16 samples were under the target. For the same reason, the relevant threshold of 100 cells/μL could not be assessed. Patients presenting with low CD4 values are expected to become less frequent as a consequence of the Test and Treat strategy [3;7]. However, providing ART to all patients in resource-limited settings is not yet operational, and POC instruments are primarily designed for such settings. Moreover, future CD4 enumerations in the future may focus on prophylaxis, which applies to thresholds of 100 to 350 cells/μl. Similarly to the previous studies, the specificity was very high, due to an average positive bias from the FACSPresto, leading to treatment a short ART initiation delay, as the LOA range for the FACSPresto was less than 100 CD4 cells/μL. Such a low LOA has been shown to have a limited effect on the clinical decision [23]. Unfortunately, still 22 patients (37% of misclassified, 9.8% of total) were misclassified with > 100 cells/μL when capillary blood was used (threshold of 350 cells/μL in Dar es Salaam). Clinicians and decision-makers should be aware of this limitation when implementing this capillary blood approach in their setting, as patients may initiate their ART (where this threshold is still applied) or their prophylaxis with co-trimoxazole too late, increasing risks of developping opportunistic infections.

Sample rejection rate was lower than in South Africa (11% versus 16%), but most invalid results were linked to an instrument failure, and not to samples with low CD4 values. The proportion of samples without a final result (6%), higher than in the Kenyan study (1.9%), may be a concern although it was still considered acceptable for health care management (should be less than 10% according to the WHO evaluation protocol) [17]. However, in a setting with high numbers of patients with very low CD4 counts, the absence of a result rather than an indication of “low value” may be a limitation of the instrument; if precise quantitative data are indeed not possible under a certain CD4 threshold, a semi-quantitative result would have been more informative in this case.

The FACSPresto instrument has been designed for use in a non-laboratory environment close to the patient and patient care. Ease of use is similar to the Pima CD4, one other CD4-dedicated POC prequalified by WHO. When comparing the performance on both intruments, the FACSPresto showed better presicion, trueness and a lower rejection rate than Pima CD4 [13;24–27]. In both cases, capillary blood did not perform as good as venous blood. Interestingly, in contrast to FACSPresto, the Pima CD4 tended to underestimate the absolute CD4 count, leading to earlier treatment initiation rather than delay.

The evaluation conditions may also be considered as a limitation of the present study. This POC instrument was assessed in well-equipped reference labs, and an evaluation outside the laboratory by non-lab personnel has not been done. However, operators (technicians) found the FACSPresto very easy to use. In addition, it has been reported that patients accept finger prick better than venipuncture for CD4 counting at the point-of-care [28]. On the contrary, the staff responsible for capillary blood collection (nurses in Antwerp and technicians in Dar es Salaam) was not used to this approach, and preferred collecting venous over capillary blood. The lack of routine experience in finger pricking may also be a reason for the observed variability in performance. When evaluating or implementing capillary blood for CD4 enumeration, enough attention should be paid to this aspect, in addition to the specific training given by the manufacturer.

Another limitation of the FACSPresto may be the absence of integrated connectivity which would be desirable to facilitate electronic reporting of results and trouble shooting. The presence of an USB port may however allow the connection of an external GPRS, and the electronic export and transfer of results.

In conclusion, overall, FACSPresto showed a very good performance when using venous and capillary blood. The instrument was considered easy to use, with minimal training and infrastructure required, making it suitable for resource-limited settings. However, users should be aware that accuracy was lower when using capillary blood, emphasizing the need for dedicated training in settings where blood collection by finger prick would be applied.

## Supporting Information

S1 File Excel file with raw data from Belgium. 1. Clinical data; 2. Adverse events; 3. Intra-assay precision; 4. Inter-instrument precision; 5. Inter-assay precision; 6. Instrument precision; 7. Trueness FACSPresto vs FACSCalibur; 8. Internal controls FACSPresto; 9. External controls Multicheck on FACSCalibur; 10. External controls 10. CD-Chex Plus BC Streck on FACSPresto.
(XLSX)

S2 File Excel file with raw data from Tanzania. 1. Adverse events; 2. Intra-assay precision; 3. Inter-assay precision; 4. Instrument precision; 5. Intra-instrument precision (operator 1); 6. Intra-instrument precision (operator 2); 7. Intra-instrument precision (operator 3); 8. Trueness data on FACSPresto; 9. Trueness data on FACSPresto; 10. CD-Chex Plus BC Streck on FACSPresto.
(XLSX)

S1 Table Precision (%CV) determined by the different operators in Dar es Salaam. For each sample (n = 4) and operator (n = 3), %CV was calculated from ten replicates on each of the three instruments (n = 30).
(DOCX)

S2 Table Precision (%CV) determined on internal controls and on stabilized blood. Mean precision (%CV, coefficient of variation) in Antwerp and in Dar es Salaam, for different controls. Internal controls are in-built controls in each FACSPresto instrument (pilot 1–4 in Antwerp, and 5–7 in Dar es Salaam). CD-Chex controls (Streck) are external stabilized blood controls with a shelf life of one month (2 lots used on each FACSPresto instrument). Multicheck controls (Becton Dickinson) are external controls with a shelf life of one month (up to 3 lots used on the FACSCalibur). * n = 51 for CD4%.
(DOCX)

S3 Table Rejection (invalid result) occurrence when reading 447 samples in agreement study. Rejection rate on 447 agreement samples on capillary and venous blood, in Antwerp and in Dar es Salaam.
(DOCX)

S4 Table Rejection (invalid result) occurrence on the different FACSPresto instruments. Rejection rate on 447 agreement samples on capillary and venous blood, according to FACSPresto instrument (pilot 1–4 in Antwerp, and 5–7 in Dar es Salaam).
(DOCX)
