# Supplementary material for: Performance of FACSPresto Point-of-Care Instrument for CD4-T Cell Enumeration in Human Immunodeficiency Virus (HIV)-Infected Patients Attending Care and Treatment Clinics in Belgium and Tanzania
Source: PLoS One. 2017 Jan 27;12(1):e0170248. doi: 10.1371/journal.pone.0170248 (PMC5271305; doi:10.1371/journal.pone.0170248)
Supplement: S1 Table — For each sample (n = 4) and operator (n = 3), %CV was calculated from ten replicates on each of the three instruments (n = 30). (DOCX) [file pone.0170248.s003.docx]

|  | Operator 1 | | Operator 2 | | Operator 3 | |
| --- | --- | --- | --- | --- | --- | --- |
|  | CD4 | CD4% | CD4 | CD4% | CD4 | CD4% |
| Sample 1 | 9,2 | 7,9 | 4,7 | 3,0 | 11,3 | 8,5 |
| Sample 2 | 4,0 | 3,6 | 6,0 | 5,2 | 4,1 | 3,5 |
| Sample 3 | 2,4 | 1,5 | 3,4 | 1,5 | 3,0 | 2,2 |
| Sample 4 | 3,4 | 2,1 | 1,2 | 1,3 | 4,5 | 2,6 |
| Mean | 4,8 | 3,8 | 3,8 | 2,7 | 5,7 | 4,2 |
