# Supplementary material for: Performance of FACSPresto Point-of-Care Instrument for CD4-T Cell Enumeration in Human Immunodeficiency Virus (HIV)-Infected Patients Attending Care and Treatment Clinics in Belgium and Tanzania
Source: PLoS One. 2017 Jan 27;12(1):e0170248. doi: 10.1371/journal.pone.0170248 (PMC5271305; doi:10.1371/journal.pone.0170248)
Supplement: S2 Table — Mean precision (%CV, coefficient of variation) in Antwerp and in Dar es Salaam, for different controls. Internal controls are in-built controls in each FACSPresto instrument (pilot 1–4 in Antwerp, and 5–7 in Dar es Salaam). CD-Chex controls (Streck) are external stabilized blood controls with a shelf life of one month (2 lots used on each FACSPresto instrument). Multicheck controls (Becton Dickinson) are external controls with a shelf life of one month (up to 3 lots used on the FACSCalibur). * n = 51 for CD4%. (DOCX) [file pone.0170248.s004.docx]

|  | **Control Low** | | | | | | **Control Normal** | | | | | |
| --- | --- | --- | --- | --- | --- | --- | --- | --- | --- | --- | --- | --- |
|  | Antwerp | | | Dar es Salam | | | Antwerp | | | Dar es Salam | | |
|  | n | CD4 | CD4% | n | CD4 | CD4% | n | CD4 | CD4% | n | CD4 | CD4% |
| **Internal control** | 208 | 0.16 | - | 131 | 0.11 | - | 208 | 0.14 | - | 131 | 0.07 | - |
| **Pilot 1 / 5** | 62 | 0.10 | - | 57 | 0.12 | - | 62 | 0.12 | - | 57 | 0.09 | - |
| **Pilot 2 / 6** | 47 | 0.25 | - | 37 | 0.14 | - | 47 | 0.28 | - | 37 | 0.05 | - |
| **Pilot 3 / 7** | 40 | 0.07 | - | 37 | 0.09 | - | 40 | 0.07 | - | 37 | 0.08 | - |
| **Pilot 4** | 59 | 0.20 | - | - | - | - | 59 | 0.10 | - | - | - | - |
| **CD-Chex control** | 105 | 7.9 | 6.2 | 52* | 9.0 | 4.8 | 102 | 3.1 | 2.1 | 53 | 3.0 | 2.6 |
| **Pilot 1 / 5** | 34 | 8.0 | 6.2 |  |  |  | 34 | 3.1 | 1.9 |  |  |  |
| **Pilot 2 / 6** | 19 | 7.4 | 5.7 |  |  |  | 19 | 4.0 | 2.5 |  |  |  |
| **Pilot 3 / 7** | 18 | 8.4 | 6.5 |  |  |  | 15 | 2.3 | 1.8 |  |  |  |
| **Pilot 4** | 34 | 7.8 | 6.4 |  |  |  | 34 | 3.0 | 2.2 |  |  |  |
| **Multicheck control** | 35 | 4.9 | 5.0 |  |  |  | 44 | 6.7 | 1.8 |  |  |  |
| **Lot 1** | - |  |  | - |  |  | 9 | 6.2 | 2.2 | - |  |  |
| **Lot 2** | 22 | 5.1 | 5.8 | - |  |  | 22 | 7.4 | 1.5 | - |  |  |
| **Lot 3** | 13 | 4.7 | 3.6 | - |  |  | 13 | 5.7 | 2.0 | - |  |  |
