# Supplementary material for: Performance of FACSPresto Point-of-Care Instrument for CD4-T Cell Enumeration in Human Immunodeficiency Virus (HIV)-Infected Patients Attending Care and Treatment Clinics in Belgium and Tanzania
Source: PLoS One. 2017 Jan 27;12(1):e0170248. doi: 10.1371/journal.pone.0170248 (PMC5271305; doi:10.1371/journal.pone.0170248)
Supplement: S3 Table — Rejection rate on 447 agreement samples on capillary and venous blood, in Antwerp and in Dar es Salaam. (DOCX) [file pone.0170248.s005.docx]

|  | **Capillary blood (Antwerp)** | **Capillary blood (Dar es Salam)** | **Venous blood (Antwerp)** | **Venous blood (Dar es Salam)** |
| --- | --- | --- | --- | --- |
| **n samples** | 200 | 247 | 200 | 247 |
| **n (%) with rejection at first reading** | 11 (5.5) | 22 (8.9) | 18 (9.0) | 31 (12.6) |
| **n (%) without final value** | 5 (2.5) | 21 (8.5) | 0 (0.0) | 14 (5.7) |
