# Supplementary material for: Performance of FACSPresto Point-of-Care Instrument for CD4-T Cell Enumeration in Human Immunodeficiency Virus (HIV)-Infected Patients Attending Care and Treatment Clinics in Belgium and Tanzania
Source: PLoS One. 2017 Jan 27;12(1):e0170248. doi: 10.1371/journal.pone.0170248 (PMC5271305; doi:10.1371/journal.pone.0170248)
Supplement: S4 Table — Rejection rate on 447 agreement samples on capillary and venous blood, according to FACSPresto instrument (pilot 1–4 in Antwerp, and 5–7 in Dar es Salaam). (DOCX) [file pone.0170248.s006.docx]

| **Instrument (capillary blood)** | **Pilot 1** | **Pilot 2** | **Pilot 3** | **Pilot 4** | **Pilot 5** | **Pilot 6** | **Pilot 7** |
| --- | --- | --- | --- | --- | --- | --- | --- |
| **n samples** | 54 | 29 | 50 | 67 | 72 | 95 | 80 |
| **n with rejection at first reading** | 2 | 1 | 6 | 2 | 5 | 5 | 12 |
| **% error** | 3.7 | 3.4 | 12.0 | 3.0 | 6.9 | 5.3 | 15.0 |
| **n with instrument failure** | 0 | 1 | 4 | 1 | 5 | 5 | 11 |
| **% error** | 0.0 | 3.4 | 8.0 | 1.5 | 6.9 | 5.3 | 13.8 |
| **Instrument (venous blood)** | **Pilot 1** | **Pilot 2** | **Pilot 3** | **Pilot 4** | **Pilot 5** | **Pilot 6** | **Pilot 7** |
| **n samples** | 54 | 29 | 49 | 68 | 72 | 95 | 80 |
| **n with rejection at first reading** | 4 | 2 | 9 | 3 | 9 | 10 | 12 |
| **% error** | 5.6 | 6.9 | 18.4 | 4.4 | 12.5 | 10.5 | 15.0 |
| **n with instrument failure** | 2 | 1 | 8 | 1 | 5 | 5 | 4 |
| **% error** | 3.7 | 3.4 | 16.3 | 1.5 | 6.9 | 5.3 | 5.0 |
